# Differential Effects of Acute (Extenuating) and Chronic (Training) Exercise on Inflammation and Oxidative Stress Status in an Animal Model of Type 2 Diabetes Mellitus

**DOI:** 10.1155/2011/253061

**Published:** 2011-11-15

**Authors:** Edite Teixeira de Lemos, Rui Pinto, Jorge Oliveira, Patrícia Garrido, José Sereno, Filipa Mascarenhas-Melo, João Páscoa-Pinheiro, Frederico Teixeira, Flávio Reis

**Affiliations:** ^1^Laboratory of Pharmacology & Experimental Therapeutics, Institute for Biomedical Research on Light and Image (IBILI), Medicine Faculty, Coimbra University, 3000-548 Coimbra, Portugal; ^2^Educational, Technologies and Health Study Center, Polytechnic Institute of Viseu, 3504-510 Viseu, Portugal; ^3^Unit of Pharmacology and Pharmacotoxicology, Faculty of Pharmacy, University of Lisbon, 1649-003 Lisboa, Portugal; ^4^Agrarian School of Viseu, Polytechnic Institute of Viseu, 3500-606 Viseu, Portugal; ^5^Rehabilitation Medicine & Sports Medicine, Medicine Faculty, Coimbra University, 3000-548 Coimbra, Portugal

## Abstract

This study compares the effects of a single bout of exercise (acute extenuating) with those promoted by an exercise training program (chronic), focusing on low-grade chronic inflammation profile and on oxidative stress status, using the obese ZDF rats as a model of type 2 diabetes mellitus (T2DM). Animals were sacrificed after 12 weeks of a swimming training program and after a single bout of acute extenuating exercise. Glycaemic, insulinemic, and lipidic profile (triglycerides, total-cholesterol) were evaluated, as well as inflammatory (serum CRP*hs*, TNF-*α*, adiponectin) and oxidative (lipidic peroxidation and uric acid) status. When compared to obese diabetic sedentary rats, the animals submitted to acute exercise presented significantly lower values of glycaemia and insulinaemia, with inflammatory profile and oxidative stress significantly aggravated. The trained animals showed amelioration of glycaemic and lipidic dysmetabolism, accompanied by remarkable reduction of inflammatory and oxidative markers. In conclusion, the results presented herein suggessted that exercise pathogenesis-oriented interventions should not exacerbate underlying inflammatory stress associated with T2DM.

## 1. Introduction

Diabetes is an increasing health problem worldwide. The core pathophysiology of type 2 diabetes (T2DM) has been attributed to the classic triad of decreased insulin secretion, increased insulin resistance, and elevated hepatic glucose production. Further mechanisms have also key relevance, including those related with the fat cell (accelerated lipolysis), the gastrointestinal tract (incretin deficiency/resistance), the pancreatic *α*-cell (hyperglucagonemia), the kidney (increased glucose reabsorption), as well as the brain (insulin resistance), now referred to as the “ominous octet” [1, 2].

Hyperglycaemia and hyperlipidaemia are key promoters of diabetes dysmetabolism, namely, through the formation of reactive oxygen species (ROS) and advanced glycation end products (AGEs), which causes cell damage and insulin resistance [3, 4]. Moreover, both of them stimulate proinflammatory cytokines, thus contributing to *β*-cell degradation, particularly due to apoptosis pathways [5]. 

Exercise and diet are cornerstones of diabetes therapy [6]. Exercise has been shown to promote beneficial effects on insulin resistance, both in humans and in rodent models of T2DM [7, 8]. A correlation between the effects of acute and chronic aerobic exercise upon oxidative stress and inflammation and the diabetic dysmetabolism has been previously described [9–17]. Prospective studies assessing the physiological and biochemical effects of physical training in human populations are complex and, therefore, a suitable animal model may provide a unique research tool for such studies. The Zucker Diabetic Fatty (ZDF) rat presents a mutation in the leptin receptor gene, becoming obese, and displaying glucose intolerance, marked insulin resistance, and hyperlipidaemia. They express overt diabetes after 8 weeks of age if fed a diet containing 6.5% fat [18]. In the prediabetic state, the male ZDF rat experiences a steady increase in basal insulinaemia and plasma-free fatty acid (FFA) levels. Hyperglycaemia develops between 8 and 10 weeks of age, leading to overt diabetes and collapsing insulin secretion [19]. This pattern is similar to the progressive loss of glucose-stimulated insulin secretion found in human T2DM and, thus, the ZDF rat is viewed as a good animal model to evaluate T2DM pathophysiology and therapeutic measures [20].

The mechanisms by which regular moderate exercise exerts its protective effect remain to be fully elucidated. One possible explanation is that there is a regular acute upregulation of anti-inflammatory and antioxidant pathways, although the evidence is yet very poor. Thus, the purpose of this study is to compare the effects of a single bout of exercise (acute extenuating) with those promoted by an exercise training program (chronic), focusing on glycaemic and lipid metabolism, but particularly on low-grade chronic inflammation profile and on oxidative stress status, using the obese ZDF rats as model of T2DM.

## 2. Materials and Methods

### 2.1. Animals and Experimental Design

Male ZDF rats (ZDF/Gmi-*fa*/*fa*) were obtained from Charles River Laboratories (Paris, France) at 6 wks of age. Rats were singly housed in opaque microisolation cages, handled daily, and kept at a constant temperature of 22-23°C in humidity-controlled rooms on a standard 12:12-h (07 : 00–19 : 00) light-dark cycle. The animals were fed with distilled water supplied *ad libitum* and rodent maintenance chow (A-04 Panlab, Barcelona, Spain) adapted to their weight (100 mg/g of weight).

Twenty-four male diabetic ZDF (fa/fa) rats and eight lean ZDF (+/+) rats were obtained at 6 weeks of age. All animals underwent 2-week acclimatization period. When aged 8 weeks, the diabetic ZDF (fa/fa) rats were randomly divided into two groups: sedentary (*n* = 16) and exercised (*n* = 8). The animals were than incorporated into the long-term study of 12 weeks, after which they were sacrificed, when aged 20 weeks of age. Before sacrifice, eight diabetic ZDF (fa/fa) rats of the sedentary group were randomly chosen for swum until exhaustion in a proper swimming tank, after which were immediately sacrificed; these animals constituted the diabetic ZDF (fa/fa) acute exercise group. The lean ZDF (+/+) animals (*n* = 8) were used as the sedentary lean control group.

### 2.2. Exercise Protocols

Each day, animals were transported to a treatment room, where exercise animals were forced to swim. The exercise group swam in a cylindrical tank with a diameter and height of 60 and 100 cm, respectively, in water at a depth of 30–45 cm. To minimize stress associated with cold or hot water exposure, water temperature was monitored and maintained at 36°C. Initially, rats swam 15 min/d (5 d/wk); the exercise protocol was gradually increased by 15 min/d until a swimming period of 1 h/d (1 wk) was attained. Thereafter, rats swam 1 h/d, 3 d/wk, for an additional period of 11 wks as described previously [21]. After 12 wks, exercised rats were sacrificed 48 hours after the last bout of exercise. The remaining sedentary rats were randomly divided in two groups, one of which was forced to swim until exhaustion. Immediately after the exhaustive bout of exercise, rats were sacrificed by decapitation. Exhaustion was defined as that point at which the animal could not remain at the water surface [22]. 

Sedentary control ZDF and lean animals were subjected to the same sampling and handling procedures as exercise training and acutely exercised animals, except they remained in their cages without food and water for the duration of the swimming exercise period.

### 2.3. Glycaemic, Insulinemic, and Lipidic Profile

Plasma glucose levels were measured using a Glucose oxidase commercial kit (Sigma, St. Louis, Mo, USA). Taking into account the variability of serum glucose levels in rats, glycosylated hemoglobin (HbA_1_c) levels were used as an index of glucose control. HbA_1_c was measured using the DCA 2000+ latex immunoagglutination method (Bayer Diagnostics, Barcelona, Spain). Plasma insulin levels were quantified by using a Rat Insulin Elisa Assay Kit (Mercodia, Uppsala, Sweden). Insulin sensitivity of individual animals was evaluated using the previously validated homeostasis model assessment (HOMA) index [23]. The formula used was as follows: [HOMA-IR] = fasting serum glucose (mg/dL) × fasting serum insulin (*μ*U/mL)/405. The values used (insulin and glucose) were obtained after an overnight of food deprivation. Serum total cholesterol (Total-Chol) and triglycerides (TGs) were analysed on a Hitachi 717 analyser (Roche Diagnostics) using standard laboratorial methods. Total-cholesterol reagents and TGs kit were obtained from bioMérieux (Lyon, France).

### 2.4. Serum Inflammatory Profile and Redox Status

Serum levels of tumour necrosis factor *α* (TNF-*α*) and adiponectin were measured by rat-specific enzyme-linked immunosorbent assay (Quantikine ELISA kits, R&D Systems, Minneapolis, USA). High-sensitive C-reactive protein (CRP*hs*) was determined by rat-specific enzyme-linked immunosorbent assay from Helica Biosystems, Inc. (Fullerton, CA, USA). All assays were performed according to the manufacturer's recommendations, in duplicate. The thiobarbituric acid reactive-species (TBARs) assay was used to assess serum products of lipid peroxidation, via malondialdehyde (MDA), according to previously described [24]. Samples were analysed at 532 nm using 1,1,3,3-tetramethoxypropane as external standard. The concentration of lipid peroxides (in MDA) was expressed as *μ*mol/L in serum. Uric acid (UA) was measured using enzymatic-colorimetric methods (Roche Diagnostics, GmbH, Mannheim, Germany).

### 2.5. Statistical Analysis

All values are reported as mean ± SEM (standard error of means). Significance level was accepted at *P* < 0.05. Data were analyzed using SPSS Statistics 18 (2009). For all parameters measured at final time, a one-way (treatment) ANOVA was used. LSD's post hoc analysis was used to determine differences between relevant mean values.

## 3. Results

### 3.1. Body Weight and Lipid Profile

The sedentary obese diabetic ZDF (*fa/fa*) rats presented significantly higher body weight (*P* < 0.001) when compared with the sedentary lean control ZDF (+/+) rats (Table 1). No significant changes on body weight gain were encountered between the exercised groups of diabetic ZDF (*fa/fa*) rats (swim trained and acute extenuating) versus the sedentary ones (Table 1). When compared with lean control, the diabetic (*fa/fa) *rats had higher Total-Chol (+175%; *P* < 0.001) and TGs (+594%; *P* < 0.001) values. These changes in biochemical indices are according to the expected because, as the uncontrolled diabetic status progresses, substantial changes in these biochemical/metabolic indices are predictable.

The diabetic ZDF (*fa/fa*) rats that underwent swim training showed significantly lower values of serum Total-Chol (+20.6%; *P* < 0.001) and TGs (+30.8%; *P* < 0.001), when compared with the sedentary counterparts. Acute extenuating exercise had no lowering effects, neither on Total-Chol nor on TGs levels (Table 1).

### 3.2. Glycaemia, HbA1c, Insulinaemia, and Insulin Resistance

As expected, the sedentary diabetic (*fa/fa) *rats showed significantly (*P* < 0.001) higher values of glucose (+359%), HbA1c (+272%), and insulin (+270%), together with higher insulin resistance (HOMA-IR), when compared with their lean sedentary counterparts (Table 1). Both groups of exercised (acute and chronic) obese diabetic ZDF (*fa/fa*) rats showed a reduction in glucose levels, when compared to the diabetic ZDF sedentary ones; nevertheless, the reduction was particularly evident in the rats submitted to acute extenuating exercise (−60.7%; *P* < 0.001). When comparing the glucose levels of the acutely exercised versus the trained ZDF rats, a significant lower value in the first was also found (−56.0%; *P* < 0.001) (Table 1). The 12-week swim training program also significantly diminished the HbA1c levels (−9.3%; *P* < 0.01) in the diabetic ZDF rats, although no changes were detected in HbA1c levels of the group submitted to the acute bout of exercise. Exercise training and acute exercise were able to significantly reduce (*P* < 0.001) insulin levels of diabetic ZDF rats (Table 1). In agreement with the above-mentioned lowering effects on glucose and insulin serum levels by exercise (training and acute) in diabetic ZDF rats, the insulin resistance, evaluated by HOMA-IR index, was significantly reduced (*P* < 0.001), in those submitted to exercise (Table 1). Nevertheless, this drop in insulin resistance was more evident in the diabetic rats submitted to the acute bout of exercise (−63.4%) than in the swim-trained diabetic ZDF rats (−13.6%).

### 3.3. Serum Markers of Inflammation

**Figure 1:**
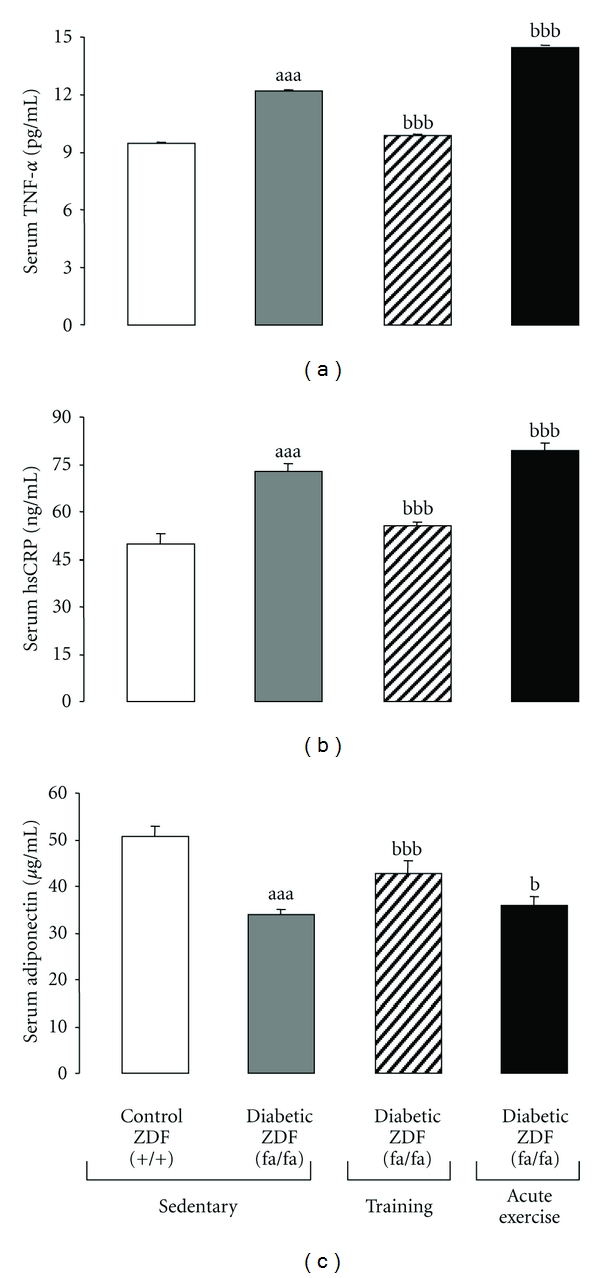
Effect of chronic moderate regular exercise (training) versus acute (extenuating) exercise on serum inflammatory markers of diabetic ZDF (fa/fa) rats: (a) TNF-alpha, (b) CRP*hs,* and (c) Adiponectin. Results are mean ± sem of 8 rats per each group. Statistically significant differences are indicated by the symbols: (a) versus sedentary control ZDF (+/+) group and (b) versus sedentary diabetic ZDF (fa/fa) group. *P* < 0.05, *P* < 0.01, and *P* < 0.001 for one, two, or three symbols.

The effects of exercise (training and acute) in systemic inflammation exhibit by sedentary diabetic ZDF rats when compared with their lean counterparts are shown in Figure 1. The sedentary diabetic ZDF rats showed significantly (*P* < 0.001) higher values of TNF-*α* and CRP*hs*, and lower of adiponectin, when compared with the sedentary control lean ZDF (+/+) rats (Figures 1(a), 1(b), and 1(c)). The 12-week swim training in the diabetic ZDF (*fa/fa*) rats showed an inflammatory lowering effect. Therefore, in the trained rats, serum levels of TNF-*α* were significantly decreased (−19.2%; *P* < 0.001), to values near to those found in the sedentary lean control (9.85 ± 0.24 versus 9.47 ± 0.09) (Figure 1(a)), which was also obtained for CRP*hs* levels (−23.7%; *P* < 0.001) (Figure 1(b)). Swimming training was also able to significantly (*P* < 0.001) raise the serum adiponectin levels in the diabetic ZDF rats versus the sedentary diabetic ZDF animals (42.63 ± 0.58 pg/mL versus 33.90 ± 1.11 pg/mL, resp.) (Figure 1(c)). Acute extenuating exercise also promote a recuperation of adiponectin (+5.8%; *P* < 0.05) levels (Figure 1(c)), but further increase of serum TNF-*α* (+18.5%; *P* < 0.001) and CRP*hs* (+8.7% *P* < 0.05) contents was obtained, when compared with the sedentary diabetic ZDF (*fa/fa*) rats (Figures 1(a) and 1(b), resp.).

### 3.4. Serum Lipid Peroxidation (TBARs) and Uric Acid Content

**Figure 2:**
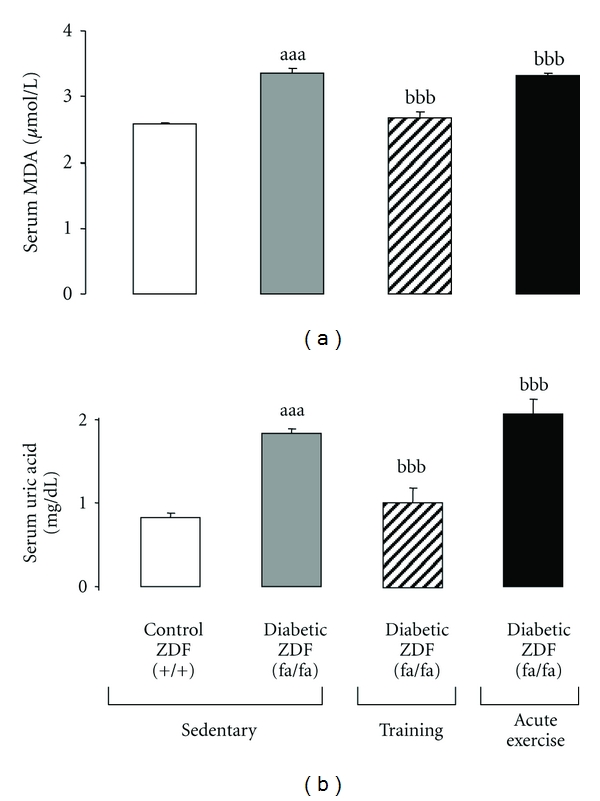
Effect of chronic moderate regular exercise (training) versus acute (extenuating) exercise on serum redox status of diabetic ZDF (fa/fa) rats: (a) lipid peroxidation (TBARs, via MDA) and (b) uric acid. Results are mean ± sem of 8 rats per each group. Statistically significant differences are indicated by the symbols: (a) versus sedentary control ZDF (+/+) group and (b) versus sedentary diabetic ZDF (fa/fa) group. *P* < 0.05, *P* < 0.01, and *P* < 0.001 for one, two, or three symbols.

The serum MDA levels, a widely accepted marker of lipid peroxidation, were significantly higher (*P* < 0.001) in the sedentary diabetic ZDF (*fa/fa*) rats when compared with the lean sedentary control ZDF (+/+) rats (Figure 2(a)). While the exercise training was able to significantly (*P* < 0.001) reduce serum MDA levels of the diabetic ZDF rats to values identical to those found in the lean control ZDF (+/+) rats, acute extenuating exercise slightly, but significantly, augmented the lipid peroxidation levels encountered in the sedentary diabetic animals (+11.13; *P* < 0.01) (Figure 2(a)). 

The high levels of uric acid presented by sedentary diabetic ZDF rats, when compared with the lean control rats (1.83 ± 0.01 mg/dL versus 0.82 ± 0.01 mg/dL), were significantly diminished in the group submitted to swim training (−44.03%, *P* < 0.001). In the diabetic group submitted to an acute extenuating bout of exercise, a slightly, but significantly, enhancement of uric acid levels was observed (+13.40%, *P* < 0.01) (Figure 2(b)).

## 4. Discussion

The effects of exercise in diabetic organism are of great interest to the medical and scientific community. While the beneficial effects of physical activity on metabolic profile are better known, the influences on mechanisms of oxidative stress and inflammation that underlie diabetes are now starting to be elucidated, in particular due to animal studies [9, 21]. Therefore, studies assessing the physiological and biochemical effects of exercise in humans are logistically and methodologically complex and we should rely on animal models submitted to laboratory protocols of exercise that mimic the training response in humans. The ZDF rat has been widely used as an animal model of human T2DM to study pathophysiological aspects, as well as therapeutic influences [25]. Swimming exercise has been also commonly used to identify the physiologic, biochemical, and molecular responses to exercise stress [26], mainly because it is a uniform type of activity that is less traumatic to animals. Thus, swimming protocols could be used to assess another less studied issue in this context: evaluate the differences between acute (extenuating) and chronic (training) on type 2 diabetes pathophysiology, including on inflammation and oxidative stress. 

In the present study, the acute bout of exercise presented a significant glucose-lowering effect in obese diabetic ZDF rats, accompanied by a reduction on insulinaemia and a decrease in insulin resistance, evaluated by HOMA-IR. These results are in agreement with other studies performed in obese rodents [27], and in T2DM individuals [28, 29]. The effect of acute exercise on glycaemia is likely due to the ability of skeletal muscle contractile activity to activate glucose transport, as this pathway appears to be normal in animal models of insulin resistance [30, 31] and in T2DM subjects [32]. Despite this amelioration in glucose metabolism by acute exercise, it was unable to induce beneficial modifications in the lipidic profile (TGs and Total-Chol). However, others have evidenced in men that fasting plasma TGs concentrations are reduced 12–24 h after a single bout of prolonged endurance exercise and until 2-3 days later [33]. 

The modifications induced by the 12-weeks swimming training protocol in ZDF rats demonstrated a delay in the development of T2DM in this animal model [21]. Király et al. obtained similar results in diabetic ZDF rats that underwent a 13-weeks protocol of forced swimming training. Additionally, in our study, the hyperinsulinaemia was partially, but significantly, corrected in the trained rats, which was accompanied by reduction of insulin resistance, given by the lower HOMA-IR value [34]. Thus, we hypothesize that swimming training was able to improve peripheral insulin resistance, even though the less action on hepatic resistance, suggesting that hyperinsulinaemia could be a reflex of insulin resistance in the liver, not improved by exercise [9, 21]. In accordance with previous studies from our group [9, 21], the results now obtained also demonstrated that aerobic exercise training improved the lipidic profile of trained diabetic ZDF rats, namely by reducing the Total-Chol and TGs contents.

It is now well established that in obesity, insulin resistance is, at least in part, mediated by cytokines produced in adipose tissue, such as TNF-*α* and IL-6 [9, 21, 35]. Exercise represents a physical stress that challenges homeostasis [36]. Therefore, a single bout of exercise is a mild physical stressor stimulus that exerts an array of effects on immune parameters [37]. The body reacts to physical activity as it does during an acute, subclinical inflammatory response to a perceived pathological insult [38]. This includes the production of TNF-*α*, an proinflammatory cytokine that induces the synthesis of other cytokines, such as IL-6 and the acute-phase reactants CRP and haptoglobin [39]. We measured TNF-*α*, because is the first molecular link between obesity and T2DM. This inflammatory cytokine is overexpressed and over-produced in the adipose tissues of rodent models of obesity and obese humans [40]. The diabetic ZDF rats presented a significantly greater circulating concentration of TNF-*α*, IL-6 and CRPhs, as well as lower concentrations of adiponectin, when compared to their lean counterparts. We also found that circulating basal levels of TNF-*α* were augmented by acute extenuating exercise. Similar studies on the literature showed controversial results. Therefore, in most of the studies, TNF-*α* does not change or presented a small increase [41], while, in contrast, Nieman et al. reported an approximately two-fold increase in TNF-*α* mRNA levels in the thigh muscle of human subjects after either 2 h resistance exercise or 3 h treadmill running [42]. Furthermore, Hamada et al. showed an elevated TNF-*α* mRNA levels in vastus lateralis muscle of young and old men 72 h after a downhill running (−16%) at 75% VO_2 max_ [43]. Binding of TNF-*α* with plasma membrane could increase vascular permeability and stimulate adhesion molecules expression on the cell surface, which allows for diapedesis and rapid infiltration of polymorphoneutrophils (PMN) into the muscle cells [44]. The invading PMN have the ability to release proteases that facilitate the degradation of cellular debris produced by muscle damage, but more importantly, they trigger a chain of cellular events that last well beyond the cessation of extenuating exercise [45]. Even thought that IL-6 is closely associated with TNF-*α* in exercise [39], in the present work we chose CRPhs as marker of chronic low-grade inflammation, because CRP is positively correlated with obesity, insulin resistance and has been shown to better predict CVD than other cytokines [46]. The results obtained by our group showed that the acute exhausting bout of swim practiced by diabetic ZDF rats augments CRPhs levels, which were already high in sedentary diabetic rats versus control lean rats. Several studies examined the acute phase response in humans [47, 48], concluding that CRP levels are increased after strenuous exercise, suggesting that this effect could be related to the type and duration of exercise, as well as with the muscle mass involved. 

Adiponectin, an adipocitokine, plays a significant role in T2DM and metabolic syndrome, due to its insulin sensitizing anti-inflammatory and anti-atherogenic properties [49]. Circulating adiponectin levels, unlike other adipokines, are reduced in obesity. Adiponectin reduces the postprandial increase in plasma FFAs and affects hepatic glucose output [50]. There is interplay between the oxidation of lipids and carbohydrates during exercise, with lower intensities associated with a greater percentage of FFAs oxidation and higher intensities with greater carbohydrate utilization; exercise intensity also affects the reliance on the form (glycogen or glucose) of carbohydrate oxidized [51]. In studies using rodent, adiponectin has been shown to regulate plasma FFAs clearance by stimulating skeletal muscle FFA uptake [52] and/or oxidation [53]. Our results showed in the diabetic ZDF rats that underwent acute strenuous exercise an augment of adiponectin levels (+5.8%; *P* < 0.05). Zeng et al. [53] showed in SD rats that a single session of treadmill exercise did not affect blood adiponectin level. In humans the majority of studies also concluded that acute exercise does not affect adiponectin concentrations in women as well as in men. Only in a graded treadmill walk/run protocol, Kraemer et al. demonstrated significant increases in adiponectin concentrations of runners that may be attributed to normal plasma volume shifts [54]. 

Since oxidative stress and reactive oxygen species (ROS) generation lead to the activation of redox sensitive pro-inflammatory transcription factors [55], we also studied the effect of exercise (chronic versus acute) on oxidative stress. Both hyperglycaemia and hyperlipidaemia are responsible for increasing the production of free radicals, which are known to accelerate the pathogenesis of T2DM, as evidenced by the glucose and lipid toxicity theories [56]. The vast majority of studies on this issue have reported increases in various oxidative stress biomarkers in several tissues following a myriad of aerobic exercise protocols [57]. Our results showed that acute extenuating exercise significantly augments the already elevated MDA levels found in the control sedentary diabetic ZDF (fa/fa) animals, vs the control lean ZDF (+/+) rats. These results suggest that the intensity and duration of the acute swimming exercise was able to exceed the antioxidant defences. The increase of UA levels in the rats that underwent acute exercise seems to corroborate these findings. The augment of UA during an acute bout of exhaustive exercise is in agreement with the mechanism referred herein of enhanced catabolism of purine nucleotides. This increase, in turn, depends on the metabolic rate, and is associated with the production of free radicals [58]. Our results corroborate those obtained by Gomez-Cabrera et al. using male wistar rats exercised in treadmill until exhaustion [59]. Mild oxidative stress induced by exercise appears to be able to reduce oxidative damage. In the current study, the diabetic ZDF rats submitted to a thrice weekly swimming training showed a decline in lipid peroxidation, in terms of MDA content. These results are in accordance with Király et al. in ZDF rats submitted to a 5-weeks swimming program (1 h/day; 5 days/wk) [34] and with other previous studies of our group [9]. The positive effects of training in ZDF rats were extended to UA. A diminution of 44% in serum levels of UA was encountered in the trained diabetic ZDF rats when compared to the sedentary diabetic animals. This improvement on the counterbalance of oxidative stress by exercise training in an animal model of T2DM was already found by others, also in animal models [60, 61].References and further reading may be available for this article. To view references and further reading you must purchase this article. 

There is evidence that pro-oxidative as well as pro-inflammatory effect of acute exhaustive exercise is counteracting by the anti-oxidative and anti-inflammatory effect of exercise training. A vicious cycle seems to be established between inflammation and ROS. Inflammation-induced tissue damage upregulates the activity of the enzyme xanthine oxidase, leading to an increase of UA and free radicals levels [62]. ROS are known to induce the nuclear transcription factor B (NF-kB) and monocyte chemoattractant protein-1 (MCP-1) and, thus, interfere with the inflammatory cytokines, increasing the systemic low grade inflammatory state. Overall, our findings support the idea that too extenuating exercise activity is harmful due to massive generation of ROS, if a body is not conditioned to minimize damage. Nevertheless, the transitory elevation of ROS when regularly repeated may induce up regulation of defense against oxidative stress and inflammation. It is, therefore, likely that mild oxidative stress is useful in retarding physiological decline associated to T2DM.

## 5. Conclusions

While chronic moderate training produces beneficial effects on diabetes metabolism, due to anti-inflammatory and antioxidant properties, the acute extenuating exercise might be deleterious, which recommends a judicious choice of exercise protocol for diabetic patients by physicians, namely, considering the type, the duration, and the intensity, which seem to differently affect diabetes pathophysiology.

## Figures and Tables

**Table 1:** Effect of chronic moderate regular exercise (training) versus acute (extenuating) exercise on body weight, glucose, HbA1c, insulin, HOMA-IR, Total-Chol, and TGs on diabetic ZDF (fa/fa) rats.

	Sedentary			Effect of training		Effect of acute exercise	
	Control ZDF (+/+)	Diabetic ZDF (fa/fa)	Effect	Diabetic ZDF (fa/fa)	Effect	Diabetic ZDF (fa/fa)	Effect
Body weight (g)	327.13 ± 2.30	425.50 ± 2.73^aaa^	*⇑*	438.00 ± 3.11^NS^	*⇔*	426.40 ± 2.32^NS^	*⇔*
Glucose (mg/dL)	133.82 ± 2.08	614.07 ± 3.84^aaa^	*⇑*	548.50 ± 15.35^bbb^	*⇓*	241.25 ± 5.22^bbb^	*⇓*
HbA1c (%)	3.36 ± 0.12	12.50 ± 0.12^aaa^	*⇑*	11.43 ± 0.19^bb^	*⇓*	12.05 ± 0.09^NS^	*⇔*
Insulin (pmol/L)	50.01 ± 0.99	184.89 ± 1.28^aaa^	*⇑*	178.77 ± 0.30^bbb^	*⇓*	172.46 ± 1.18^bbb^	*⇓*
HOMA-IR	2.38 ± 0.98	40.36 ± 2.02^aaa^	*⇑*	34.86 ± 1.30^bbb^	*⇓*	14.79 ± 1.02^bbb^	*⇓⇓*
Total-Chol (mg/dL)	75.91 ± 1.17	208.83 ± 1.37^aaa^	*⇑*	165.88 ± 2.13^bbb^	*⇓*	210.25 ± 0.90^NS^	*⇔*
TGs (mg/dL)	60.23 ± 0.71	418.00 ± 3.19^aaa^	*⇑*	289.38 ± 0.87^bbb^	*⇓*	417.00 ± 1.17^NS^	*⇔*

Results are mean ± sem of 8 rats per each group. Statistically significant differences are indicated by the symbols: (a) versus sedentary control ZDF (+/+) group and (b) versus sedentary diabetic ZDF (fa/fa) group. *P* < 0.05, *P* < 0.01, and *P* < 0.001 for one, two, or three symbols.
